# Marine Pyrrolocarbazoles and Analogues: Synthesis and Kinase Inhibition

**DOI:** 10.3390/md7040754

**Published:** 2009-12-01

**Authors:** Sébastien Deslandes, Stefan Chassaing, Evelyne Delfourne

**Affiliations:** Laboratoire de Synthèse et Physicochimie de Molécules d’Intérêt Biologique, UMR CNRS 5068, Université Paul Sabatier, 118 route de Narbonne, 31062 Toulouse Cédex 9, France; E-Mails: deslandes@chimie.ups-tlse.fr (S.D.); Chassaing@chimie.ups-tlse.fr (S.C.)

**Keywords:** granulatimide, isogranulatimide, pyrrolocarbazole, indolocarbazole, kinase inhibitor

## Abstract

Granulatimide and isogranulatimide are alkaloids obtained from marine sources which have been shown to inhibit cell-cycle G2-checkpoint, targeting more particularly checkpoint 1 kinase (Chk1). At a structural level, they possess a characteristic pyrrolocarbazole framework also shared by the well-known rebeccamycin and staurosporine microbial metabolites which have been described to inhibit topoisomerase I and diverse kinases, respectively. This review reports precisely on the synthesis and kinase inhibitory activities of pyrrolocarbazole-based analogues of granulatimide.

## 1. Introduction

Due to their key function in the phosphorylation of proteins, kinases act pivotally in signal transduction as well as in other cellular processes including metabolism, transcription, proliferation, apoptosis, differentiation and cell cycle progression [1]. With approximately 518 members encoded so far in the human genome, one thinks that at least one kinase is involved in every signal transduction pathway. As a result, kinases appear today as one of the most investigated classes of proteins for drug discovery.

**Figure 1.**
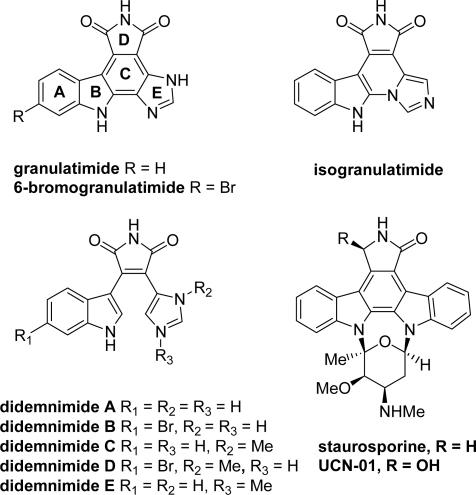
Marine pyrrolocarbazoles and related natural compounds.

Small molecule kinase inhibitors have thus emerged both as promising molecules for use in cancer therapy and as experimental tools for understanding the physiological role of these enzymes [2–9]. In this way, granulatimide and isogranulatimide, two naturally occurring alkaloids isolated from the marine ascidian *Didemnum granulatum*, were shown to be potent and selective inhibitors of Chk1 (IC_50_ values are 0.25 and 0.1 μM, respectively), Chk1 being a key kinase of cell-cycle G2 checkpoint (Figure 1) [10,11].

It is worth recalling here that the combination of a DNA damaging agent with a G2 checkpoint inhibitor constitutes a recent and attractive chemotherapeutic approach for cancer treatment. Indeed, cells respond to DNA damage by activating feedback mechanisms called checkpoints that temporarily delays the cell cycle progression and allows for DNA repair [12]. DNA damage triggers ATM and ATR protein kinases which activate Chk1 and Chk2. These checkpoint kinases, in turn, inactivate CDC25 and prevent Cdc2 activation resulting in cell cycle arrest [13]. A majority of human cancers completely lack a G1 checkpoint because of mutation of the p53 tumor suppressor gene and many cancer cells have a partially defective G2 checkpoint. Accordingly, combination of a DNA damaging agent with a G2 checkpoint inhibitor might promote cell death, by selective killing of p53-mutated tumor cells.

From a structural point of view, granulatimide and isogranulatimide possess a pyrrolocarbazole framework (*i.e.*, heterocyclic system composed of units A, B, C and D) bearing a fused imidazole heterocycle (unit E, Figure 1). Other structurally related pyrrolocarbazoles, such as staurosporine or UCN-01, were also shown to be potent but non selective inhibitors of Chk1. In terms of kinase specificity, it is noteworthy that granulatimide and isogranulatimide also inhibit Cdk1 and GSK-3β, but show less potent inhibitory activity on several other protein kinases [14]. During the last decade, large structure-activity relationship studies were carried out on these compounds, which led to more potent and more selective molecules. Whereas a previous account by Hénon *et al.* was devoted to pyrrolocarbazoles as Chk1 inhibitors [15], the present review will update and enlarge this topic, focusing on synthesis and kinase inhibition profile of analogues of the marine metabolites.

## 2. Marine-Related Metabolites and Positional Analogues

Granulatimide and isogranulatimide were first described together with didemnimide E from the ascidian *Didemnum granulatum* [10,16] (Figure 1). Isogranulatimide was also found in the Caribbean ascidian *Didemnum conchyliatum* [17] from which didemnimides A-D were isolated before [18]. Later on, reinvestigation of the extracts allowed the isolation of a new related alkaloid, *i.e.*, 6-bromogranulatimide [19]. More recently, Seleghim *et al.* have addressed the question of the biosynthetic source of the two metabolites granulatimide and isogranulatimide by localizing them within the ascidian. They demonstrated that granulatimide is stored in *Didemnum granulatum* tunic bladder cells [20].

**Scheme 1.**
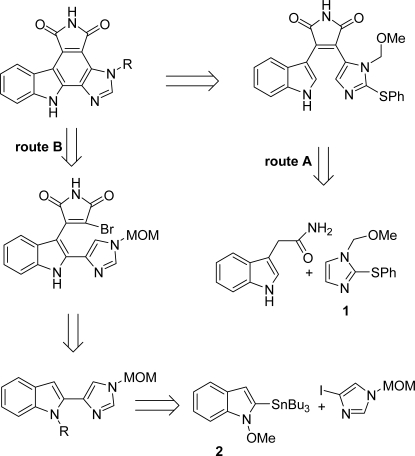


Since granulatimide and isogranulatimide have been shown to be abrogators of the cell-cycle G2-M phase checkpoint, two efficient synthetic routes have been developed for these compounds (Scheme 1). The first one by Piers *et al.* proceeded via their putative biosynthetic precursor didemnimide A and was based on the condensation of the substituted imidazole **1** with indole-3-acetamide (route A) [21]. The other one proposed by Yoshida *et al.* relied on a key Stille coupling reaction between stannylindole **2** and 4-iodoimidazole in the presence of PdCl_2_(PPh)_3_ [22] (route B).

**Figure 2.**
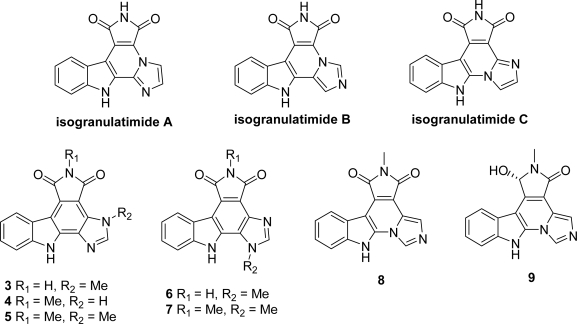
Marine-related metabolites and positional analogues of granulatimide.

The synthesis of positional analogues of granulatimide and isogranulatimide were also reported, mainly modified at the level of the imidazole ring. The first derivatives were synthesized by Piers *et al.* who prepared isogranulatimide A-C and 17-methyl granulatimide **3** by using the synthetic route they defined for the natural product [21] (Figure 2). Among them, the most interesting compounds isogranulatimide B and isogranulatimide C inhibited the G2 checkpoint with IC_50_ values of 1 and 6 μM, respectively and Chk1 with IC_50_ of 2.3 and 0.65 μM respectively.

Compound **3** was also published by Yoshida *et al.* together with 10-methylgranulatimide **4** and the disubstituted 10,17-dimethylgranulatimide **5** [22]. One year later, the same group proposed the positional isomers of the two last ones (**6** and **7** respectively) [23]. 10-methyl isogranulatimide (**8**) which was proven about 15-fold less potent than granulatimide on the G2 checkpoint assay and the 9-hydroxylated analog **9**, which did not exhibit a significant activity were also prepared [13].

## 3. Modification of the Indole Subunit (Units A and B)

The main modifications of the indole moiety consisted of: (i) introduction of substituents, (ii) replacement of this cycle by an azaindole unit, (iii) introduction of a glycosyl moiety attached to the indole nitrogen [15]. All of these compounds were proved to be poor Chk1 inhibitors, the presence of a methyl group on the imide nitrogen may be contributing to this negative result [24].

## 4. Replacement of the Imidazole Ring (Unit E)

### 4.1. By a pyrrole or by a second maleimide moiety

**Figure 3.**
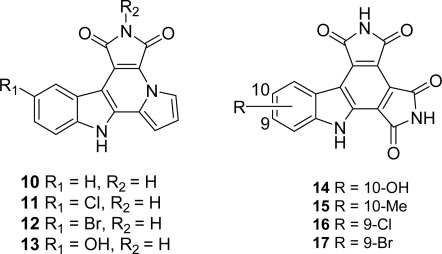
Granulatimide analogues with a pyrrole or a second maleimide moiety replacing the imidazole ring.

Most of the analogues of granulatimide and isogranulatimide reported so far result from replacement of the imidazole heterocycle by other heterocycles. Among them, both pyrrole derivatives and compounds in which the imidazole ring was replaced by a second maleimide moiety were investigated. As these compounds were intensively recapitulated in Hénon’s review [15], we just report here those which gave the most interesting results regarding their inhibitory activity toward Chk1 and their *in vitro* cytotoxicities toward four tumor cell lines including L1210, DU145, A549 and HT29 (Figure 3).

In the pyrrole series, compounds **10** to **13** exhibit stronger Chk1 inhibitory activities than granulatimide and isogranulatimide. Moreover, compounds **11** and **12** showed a significant selectivity for Chk1 when evaluated toward a large panel of kinases. There was no correlation between Chk1 inhibitory activities and cytotoxicities, the compounds exhibiting cytotoxicities in the same range than isogranulatimide. It was also proven in this study that in contrast with UCN-01, the new compounds did not bind to DNA [25].

In the second series, compound **14** and **15** exhibited IC_50_ values of 2 and 8 nM, respectively which makes them more potent Chk1 inhibitors than the natural products. The cytotoxicities toward the tumor cell lines tested including L1210, DU145, A549, HCT116 and HT29 were moderate in the case of all studied compounds except for compounds **15**, **16** and **17** [26].

### 4.2. By a non aromatic five or six-membered-ring moiety

**Scheme 2.**
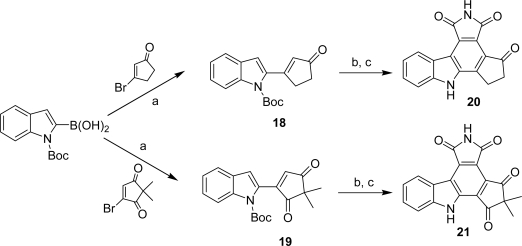
(a) Pd(PPh_3_)_4_, benzene, EtOH, Na_2_CO_3_.(c) maleimide, toluene, sealed tube, 3 days. (c) DDQ, dioxane, rt, 48 h.

**Scheme 3.**
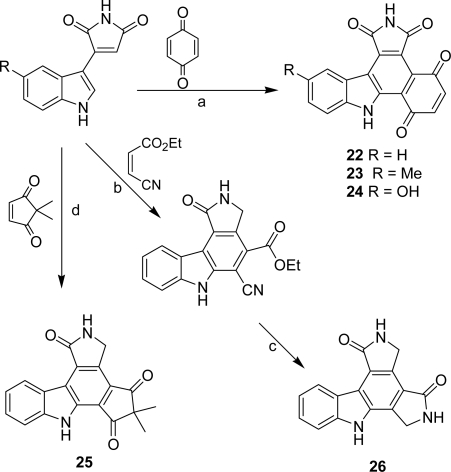
(a) toluene, sealed tube, 140 °C, 12 h. (b) (1) toluene, reflux 5 days, (2) DDQ, dioxane, reflux, 20 h. (c) (1) NaH, THF TBDMSCl, (2) H_2_, DMF, Raney nickel, 7 days. (d) toluene, 140 °C, sealed tube, 7 days.

A series of pyrrolocarbazoles **18**–**26** in which the imidazole heterocycle has been replaced by a five- or a six-membered ring carbocycle bearing one or two carbonyl functions have been published by Conchon *et al.* [27,28]. The compounds of the series were obtained either on the basis of a Suzuki coupling between 2-*N*-Boc-indoleboronic acid and 3-bromocyclopent-2-enone or 4-bromo-2,2-dimethylcyclopent-4-ene-1,3-dione followed by a Diels-Alder reaction of the adducts with maleimide (Scheme 2) or via a Diels-Alder reaction between 3-indolylmaleimide or 5-substituted-3-indolylmaleimide and different dienophiles such as cyclopent-2-enone, cyclohex-2-enone, hydroquinone, 2,2-dimethylcyclopentenedione and ethyl *cis*-β-cyanoacrylate (Scheme 3).

Among the most potent Chk1 inhibitors in this group, quinone **24** and compound **26** with a lactam D ring, exhibited IC_50_ values of 27 and 24 nM, respectively. This study showed that the d-heterocycle can be replaced by a carbocycle without loss of Chk1 activity. In addition, no parallel was observed between the cytotoxicities and the Chk1 inhibitory activities.

**Scheme 4.**
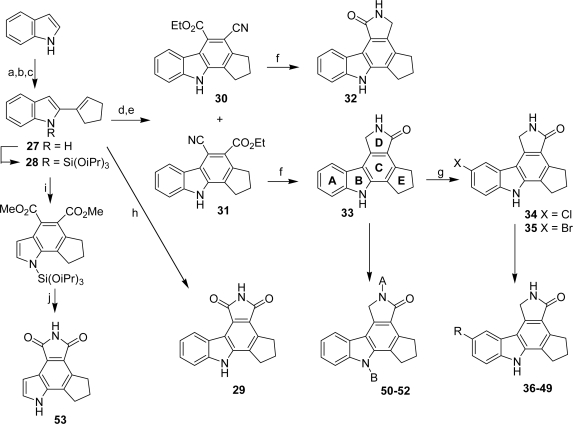
(a) *n*-BuLi, THF, −78 °C to 20 °C, then CO_2_(g). (b) *t*-BuLi, THF, −78 °C to 20 °C, then cyclopentanone. (c) 2M HCl, acetone, rt. (d) *cis-*EtO_2_CCH=CHCN, PhCl, 125 °C. (e) DDQ, toluene, 60 °C. (f) H_2_, Ra-Ni, DMF, rt. (g) NCS or NBS, DMF, rt. (h) maleimide, tetrachloroquinone, neat, 190 °C. (i) dimethyl acetylenedicarboxylate, 150 °C, 64 h. (j) (1) 10N NaOH in EtOH, reflux, 3 h, (2) Ac_2_O, 73 h, (3)(TMS)_2_NH/MeOH, DMF, 73 °C, 4 h.

Another pyrrolocarbazole series including compounds **29**–**53** related to granulatimide was identified as potent poly(ADP-ribose) polymerase-1 (PARP-1) inhibitors. This enzyme is a nuclear enzyme that catalyzes the synthesis of poly(ADP-ribose) chains from NAD^+^ in response to single-strand DNA breaks as part of the DNA repair process [29,30]. The synthesis of compounds is shown in Scheme 4.

The key diene **27** was prepared by sequential deprotonation of indole with *n*-BuLi, followed by treatment with carbon dioxide. Removal of excess CO_2_ and treatment with *t*-BuLi and cyclopentanone gave the tertiary alcohol which was dehydrated with hydrochloric acid to give **27**. Diels-Alder reaction of **27** with maleimide followed by DDQ oxidation gave the pyrrolocarbazole **29** whereas the interaction of **27** with *cis*-ethyl-3 cyanoacrylate in chlorobenzene led to a mixture of two nitrile-ester regioisomers which were separated. Subsequent DDQ oxidation of the so-obtained cycloadducts gave the corresponding derivatives **30** and **31**. Reduction of the nitrile function and spontaneous cyclization gave lactams **32** and **33**, respectively. Compound **33** was then submitted to diverse reaction conditions to give various analogues **34**–**52**. It is worth noting that the analog **53**, which did not feature the pyrrolocarbazole skeleton, was obtained by using a similar sequence of reactions. All the compounds prepared in this study were evaluated as inhibitors of recombinant human PARP-1. The results for compounds **29**–**53** reported in Table 1 are not exhaustive.

For example, different structures comprising different moieties instead of the cyclopentyl cycle E were also studied but they did not exhibit any inhibitory activity against the enzyme suggesting that this cycle is required for potency and fits into a catalytically active steric pocket of the enzyme. Data in Table 1 show an essential H-bonding interaction of both indole and lactam NH-groups within the active site of the enzyme. An open pocket near the active site corresponding to the 3 and 4 positions of the carbazole template allowed development of a SAR around this region, nevertheless this B ring is not required for activity.

### 4.3. By another aryl or heteroaryl ring moiety

**Scheme 5.**
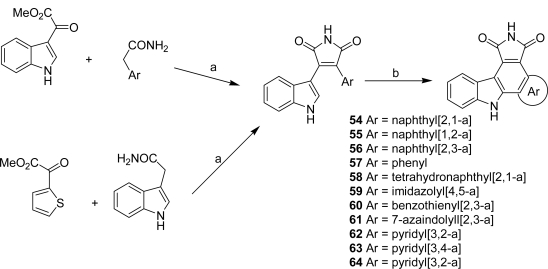
(a) *t-*BuOK, THF, 0°C to rt. (b) benzene, hν, reflux or dioxane, DDQ, hν, reflux or acetone, hν, 35 °C or Pd(OAc)_2_, AcOH.

Different analogues of granulatimide modified at the imidazole unit by an other aryl or heteroaryl ring have been proposed. In order to define new Cyclin D1-CDK4 inhibitors, Lilly Research Laboratories investigated a first series of analogues **54**–**64** [31] (Scheme 5).

Cyclin-CDK complexes regulate the progression of cells through the cell cycle, a G1-phase role being suggested for d-type cyclins through association with CDK4 and CDK6. Since abberations in CDKs and their regulators have been found in a large percentage of human tumors, inhibitors of these cyclin-CDK complexes might have a broad range of therapeutic applications in cancer. The preparation of the compounds was accomplished following Faul’s general synthetic route to access the 3-heteroaryl-4-indolyl maleimides which were cyclized either by using APTS as catalyst or by oxidative photochemistry [32,33]. Their inhibitory activity against cyclin D1-CDK4 was evaluated. From all the compounds, the naphtyl[2,1-a] derivative **54** was proven to be a potent and selective inhibitor (IC_50_ = 45 nM).

**Figure 4.**
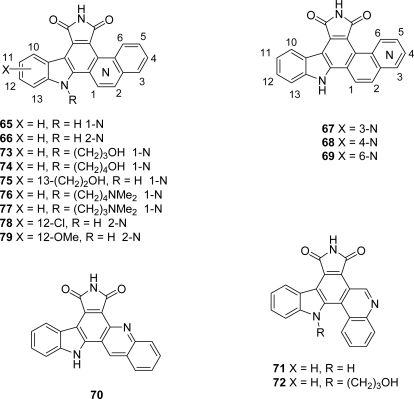
Granulatimide analogues with different aromatic moieties instead of the imidazole ring.

**Scheme 6.**
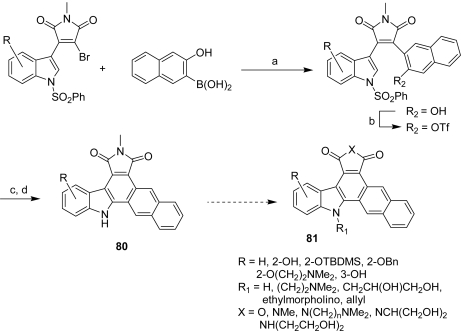
(a) Pd(OAc)_2_, K_2_CO_3_, dioxane/H_2_O, 100 °C. (b) Tf_2_O, Et_3_N, CH_2_Cl_2_. (c) Pd(OAc)_2_, PPh_3_, Bu_4_NCl, NaOAc, dioxane, 100 °C. (d) Bu_4_NF, THF, reflux.

From a second series **65**–**79**, in which the [3,4-*c*]carbazole core was fused with a quinolyl or isoquinolyl moiety, compound **68** was found to be the highest potent D1-CDK4 inhibitor with an IC_50_ of 69 nM (Figure 4). This last compound inhibited tumor cell growth, arrested tumor cells in G1-phase and inhibited pRb phosphorylation [34]. In 2005, Routier *et al.* were interested in pyrrolocarbazole-fused naphthalenes and reported an efficient route to these family of compounds **81** involving palladium-catalyzed reactions to prepare them (Scheme 6) [35].

**Figure 5.**
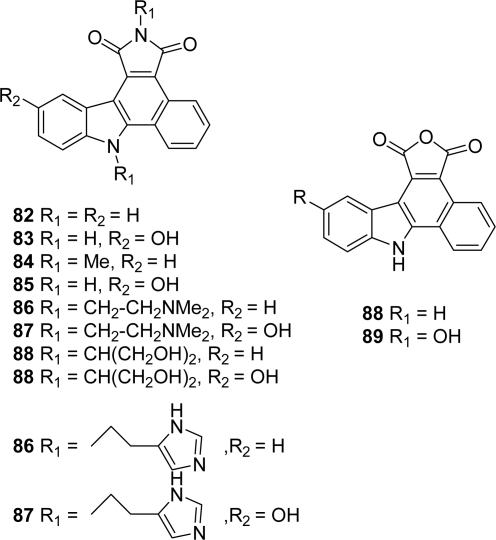
Phenylpyrrolocarbazoles proposed by Routier *et al*.

The introduction of a 2-hydroxynaphthyl group on 2-bromo-3-indolomaleimide was performed using a Suzuki or Stille coupling reaction with adequate palladium catalysts. The activation of the naphthyl as a naphthyl triflate was realized before the central six-membered ring was obtained through an elegant intramolecular Heck reaction. Different related compounds were prepared either by functionalizing the free indolic nitrogen of **80**, or by performing substitutions on the maleimide group. Some of these molecules showed marked cytotoxicity toward cancer cells including L1210, DU145 and HT29 with IC_50_ values in the sub-micromolar range. DNA binding likely contributes to the antiproliferative activity of the most cytotoxic compounds. However, no kinase inhibition was detected in this group of compounds. A second series **82**–**89** in which the imidazole ring was replaced by a phenyl group and including indolic substitution and maleimide variations was proposed by the same group [36] (Figure 5).

**Figure 6.**
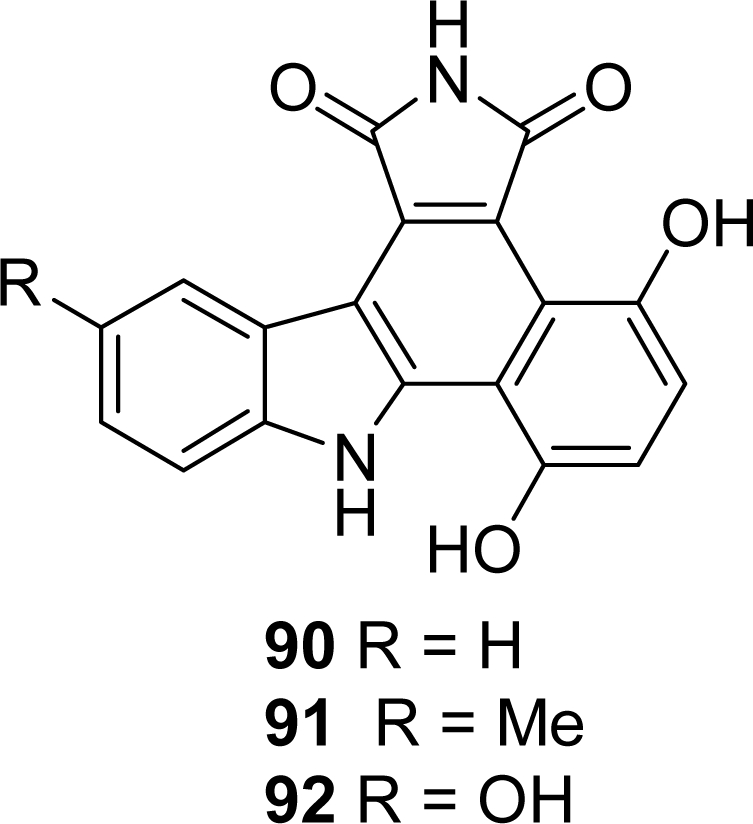
Dihydroxyphenylcarbazoles.

The compounds were prepared according to the previously described synthetic scheme, the final intramolecular key Heck-type reaction being carried out with either a triflate or a brominated derivative. Several compounds showed a marked cytotoxicity against CEM human leukemia cells with IC_50_ values in the 10–100 nM range. Although cell cycle analysis, topoisomerase I inhibition, interaction with DNA and inhibition of CDK activity were evaluated, the exact molecular targets of these molecules remain undiscovered. In their work to define new checkpoint kinase inhibitors, Conchon *et al.* were also interested in dihydroxy-phenylcarbazoles **90**–**92** which were prepared by reduction of the corresponding quinonic compounds **22**–**24** [27] (Figure 6).

**Figure 7.**
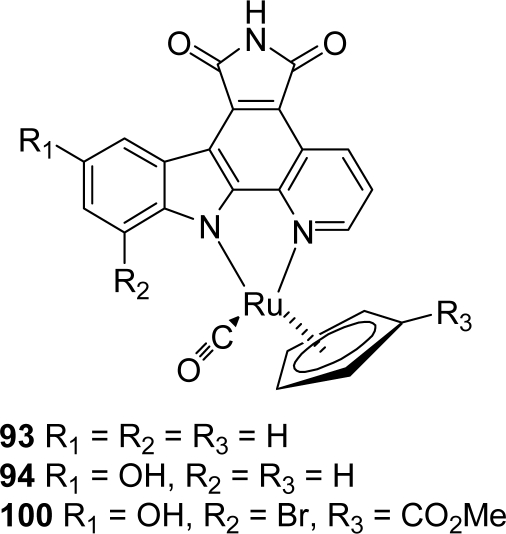
Pyridopyrrolocarbazole half-sandwich ruthenium complex.

These derivatives were shown to be potent inhibitors of the enzyme (IC_50_ 0.311, 0.161 and 0.023 μM, respectively) exhibiting Chk1 inhibitory properties very close to their analogues **22**–**24**. In 2004, during the course of their studies for developing ruthenium complexes that target the ATP-binding site of protein kinases, Meggers and his co-workers were interested in a granulatimide analogue **93** in which the imidazole ring has been replaced by a pyridinic ring (Figure 7). This cyclopentadienyl half sandwich ruthenium complex was tested as a racemic mixture against a panel of protein kinases and was thus identified as an extremely potent inhibitor for GSK-3 (IC_50_ of 3 nM for GSK-3α and 10 nM for GSK-3β), being 15,000 times more active than the ligand itself [37]. It is worth precising here that GSK-3 (glycogen synthase kinase-3) has been shown to be a key component of a diverse range of cellular processes including the regulation of glycogen metabolism or signal transduction in the insulin and wnt signal pathways [38].

**Scheme 7.**
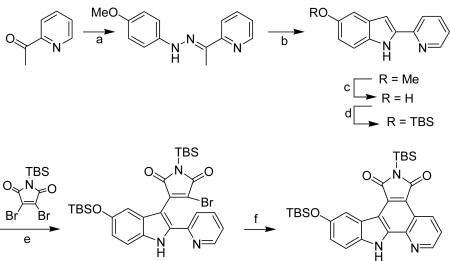
(a) 4-methoxyphenylhydrazine.HCl, *t-*BuOH, reflux, 4 h. (b) trimethylsilylpolyphosphate, 120°C, 18h. (c) BBr_3_, CH_2_Cl_2_, – 60 °C, then r.t., overnight. (d) (1) Hünig's base, DMF,0 °C, 40 min, (2) TBSOTf, 0 °C, 1 h. (e) (1) LiHMDS, THF, – 15 °C, 45 min, (2) maleimide derivative in THF, – 15 °C for 15 min, (3) r. t. for 45 min. (f) hν, pyrex filter, MeCN, 3 h.

They also reported another ruthenium complex **94** which is a highly potent inhibitor for the enzyme and demonstrated that this compound can switch on the wnt signal transduction pathway inside living cells and in *Xenopus* embryos (wnt is a secreted glycoprotein which initiates the phosphorylation of β-catenin) [39]. Later on, they performed two synthetically routes to pyrrolocarbazole-fused pyridines including either an oxidative or nonoxidative photocyclisation step, the second one being specially useful for the preparation of such analogs [40] (Scheme 7).

**Scheme 8.**
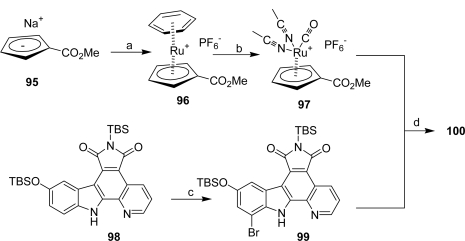
(a) [(C_6_H_6_)RuCl_2_]_2_. (b) (1) hν, MeCN, (2) CO.(c) PhMe_3_NBr_3_, rt. (d) (1) K_2_CO_3_ (2) TBAF.

In an attempt to improve the selectivity profile for GSK-3, this group achieved a simple structure-activity relationship study, starting by modifying the indole moiety and the cyclopentadienyl ligand of the half sandwich scaffold **93**. The synthesis of one compound is shown in Scheme 8.

Synthon **99**, obtained by regioselective bromination of the pyridocarbazole ligand **98** was reacted in presence of K_2_CO_3_ with cyclopentadienyl derivative **97**, itself prepared in two steps from (methylcarbonyl)cyclopentadienyl sodium **95**. Removal of the TBS group of the adduct yielded ruthenium complex **100**. After resolution of the racemic mixture, compound (*R*)-**100** was identified as the most selective derivative of GSK-3 (IC_50_ of 0.35 nM for GSK-3α and 0.55 nM for GSK-3β) against a panel of 57 protein kinases [41].

**Scheme 9.**
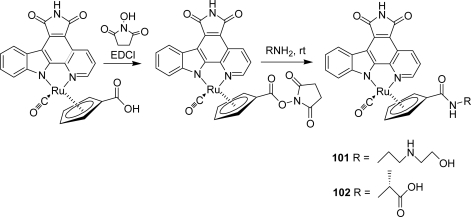


An efficient method involving an organoruthenium compound bearing a N-succinimidyl ester at the cyclopentadienyl moiety was then developed for the rapid modification of the cyclopentadienyl moiety of ruthenium half sandwich protein kinase inhibitors. The quenching of this activated ester with different amines led to the identification of Pim-1 and GSK-3 inhibitors with improved potencies and selectivities, respectively, compounds **101** and **102** [42] (Scheme 9).

**Scheme 10.**
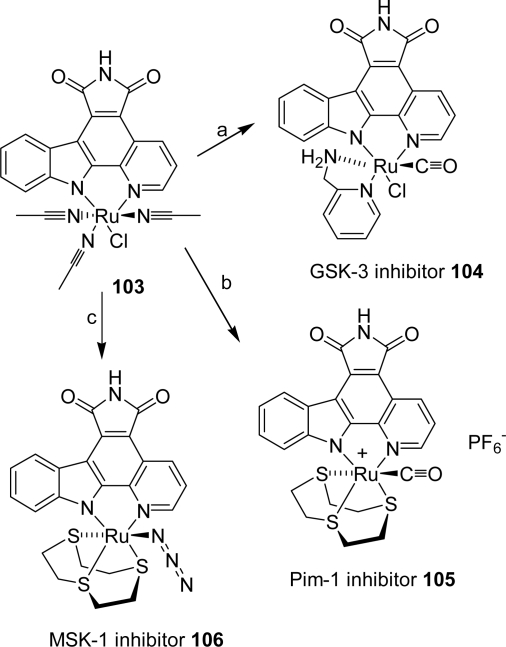
(a) CO-saturated DMF at 75 °C for 1.5 h then addition of 1 equiv of 2-aminomethylpyridine, 95 °C, 1 h. (b) (1) 1 equiv of 1,4,7-trithiacyclononane, DMF, 80 °C, 45 min (2) CO-saturated DMF, 95 °C, 2 h. (c) (1) 1 equiv of 1,4,7-trithiacyclononane, DMF, 80 °C, 1 h, (2) addition of 1 equiv of NaN_3_, 90 °C, 1 h.

A strategy that allows a rapid scanning of ligands around the ruthenium center in the search for ligand spheres that are complementary in shape and functional group presentation to ATP binding sites of individual protein kinases was presented [43]. Following this approach, octahedral ruthenium complexes **104**–**106**, prepared from precursor **103** were identified as potent inhibitors for the protein kinases Pim-1, MSK-1 and GSK-3α (Scheme 10).

**Figure 8.**
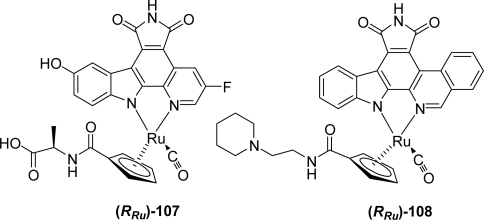
Fluoropyrido- and isoquinolino-pyrrolocarbazole half-sandwich ruthenium complex.

In 2008, Meggers’s group reported compound (*R_Ru_*) **107** containing a fluorine substituent on the pyridinic ring, to be an extremely high-affinity GSK-3 inhibitor (IC_50_ < 0.04 nM) (Figure 8) [44]. This compound was proven to perfectly complement the shape of the ATP-binding site making it one of the most potent protein kinase inhibitors reported to date.

More recently, the organoruthenium complex **108** in which the pyridinic ring has been replaced by an isoquinolic ring was proposed by Anand *et al.* to be a potent and selective Mammalian Sterile 20 kinase (MST-20) inhibitor [45]. This enzyme is a proapoptotic cytosolic kinase that plays an important role in diverse biological processes including the cellular response to oxidative stress.

### 4.4. By a second indole ring

Granulatimide analogues in which the imidazole moiety is replaced by a second indole ring are compounds also structurally very close to rebeccamycin and staurosporine antibiotics. These two compounds, isolated, respectively, from cultures of *Saccharotrix aerocologines* [46] and *Streptomyces* [47,48] differ by the sugar linked to only one indole nitrogen in rebeccamycin which contains also an imide function instead of amide function in the upper heterocycle. From a biological point of view, these disparities seem crucial in the sense that the target of these microbial metabolites are quite different. Rebeccamycin has been demonstrated to inhibit topoisomerase I, by stabilizing the enzyme-DNA interaction via a “cleavable complex”, but appeared to be inactive against PKC and PKA [49]; on the contrary, staurosporine is a non selective kinase inhibitor without activity against topoisomerases [50–53]. Large structure-activity relationship studies were carried out on these pyrrolocarbazole-fused indoles which were previously reviewed [54,55]. In the following, only works realized since 2003, will be treated.

**Figure 9.**
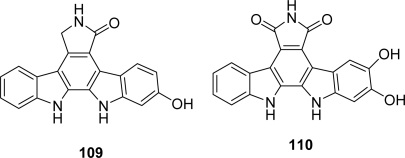
New natural indolocarbazole analogues.

New rebeccamycin-related metabolites issued from natural source have been described including 6-hydroxystaurosporine **109** and 5,6-dihydroxyarcyriaflavin A **110** which were isolated from field-collected fruit bodies of a myxomycetes *Lycogala epidendrum* [56] (Figure 9).

**Figure 10.**
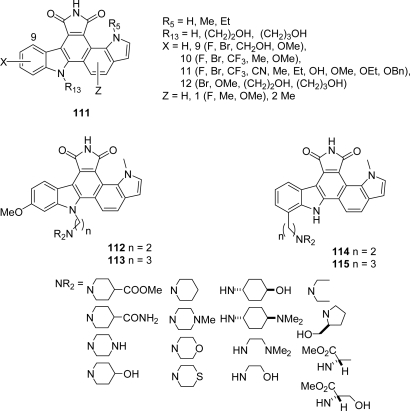
Indolo[6,7-*a*] pyrrolo[3,4-*c*]carbazoles.

These compounds showed interesting cytotoxicity against HeLa, Jurkat, and vincristine resistant KB/VJ300 cells, compound **109** also inhibiting protein tyrosine kinase activity. In continuation of their work devoted to the design of potent and selective cyclin D1/CDK4 inhibitors, workers at Lilly Research Laboratories investigated a series of [6,7-a]pyrrolo[3,4-c]carbazoles **111** substituted on the indolic nitrogens or on the indolic rings as well as other series **112**–**115** bearing different groups at N13 and C12 positions to improve aqueous solubility [57] (Figure 10). These compounds were prepared according to Faul’s synthetic scheme requiring new methods to access to 7-substituted indole acetamides and *N*-methyl(indol-7-yl)oxoacetates [58].

**Figure 11.**
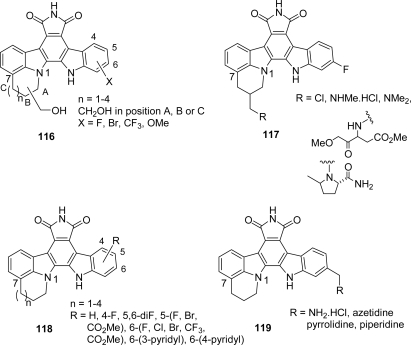
1,7-Annulated indolocarbazoles.

In addition to their potent CDK activity, the compounds displayed antiproliferative activity against two human cancer cell lines, *i.e.*, HCT-116 and H460. These inhibitors also effected strong G1-arrest in these cell lines and inhibited Rb phosphorylation consistent with inhibition of cyclin D1-CDK4. In continuous effort to optimize pharmacokinetic properties of these inhibitors, the pharmaceutic group went on exploring four series **116**–**119** of novel analogues by introducing a 1,7-annulated ring in one of the indole moiety [59,60] (Figure 11).

The compounds were obtained by submitting different 1,7-annulated indolyl-3-glyoxylates to Faul’s procedure. They all exhibited potent inhibitory activity against cyclin D1-CDK4 and good antiproliferative activity against HCT-116.

**Figure 12.**
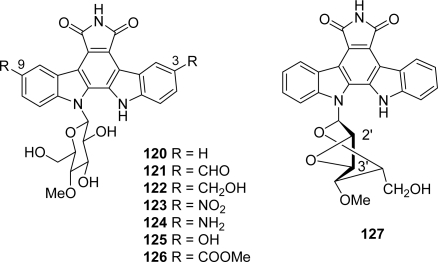
Indolocarbazole analogues substituted in 3,9-positions and 2’,3’-epoxyderivative **127**.

In the course of structure-activity relationship studies, Prudhomme and co-workers prepared by semi-synthesis from rebeccamycin, new indolocarbazole analogues **120**–**126** substituted in 3,9-positions on the indolecarbazole framework along with the 2’,3’-epoxyderivative **127** (Figure 12) [61].

The antiproliferative activities were assayed for these compounds against nine tumor cell lines and the effects on the cell cycle of murine leukaemia L1210 cells was examined. Their topoisomerase I inhibitory activity both with their activity toward three kinases including PKCζ, CDK1/cyclin B and CDK5/p25 were also evaluated. Among the different compounds of these series, the diphenol **125** was the most efficient toward CDK1/cyclin B and CDK5/p25 and appeared also to be a DNA-binder and a topoisomerase I poison. All these activities likely accounted for its cytotoxic potential.

Another series of rebeccamycin analogues **128**–**145** fluoro-substituted in the same 3,9 and/or 2,10-positions of the carbazole core was studied by Balasubramanian *et al.* with respect to their topoisomerase I activity, cytotoxicity, selectivity and *in vivo* antitumor activity [62] (Table 2).

**Scheme 11.**
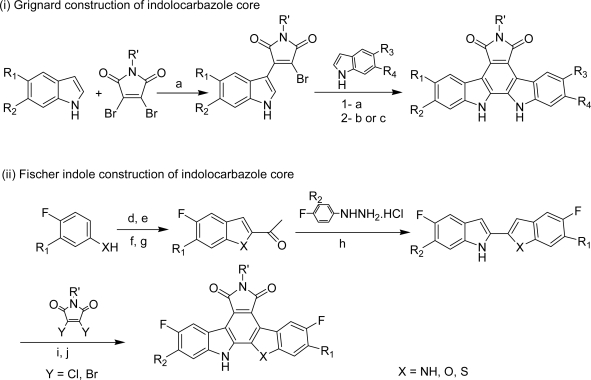
(a) 3M EtMgBr in THF, PhH, 80 °C. (b) DDQ, APTS, PhH, 80 °C. (c) hν, cat. I_2_, air, PhH, 80 °C. (d) NaOEt, EtOH, ClCH_2_CH(OEt)_2_, rt. (e) PPA, PhCl, 100 °C. (f) *n*BuLi, CH_3_CHO, THF/Et_2_O (1/1), −78 °C to 0 °C. (g) PCC, CH_2_Cl_2_, celite, rt. (h) EtOH, NaOAc, 75 °C. (i) 3M EtMgBr in THF, THF. (j) hν, EtOH, dioxane, 100 °C

The different fluorinated cores were obtained either via a stepwise addition of an appropriately substituted indole-based Grignard reagent (e.g., **128**–**135**, **140**–**142**) to a dihalomaleimide followed by oxidative cyclization or by using the Fischer indole process as an attractive alternative for the preparation of both benzofurane- and benzothiophene-fused pyrrolocarbazole analogs **136**–**139** and **143** (Scheme 11).

The introduction of the sugar moiety was finally realized using well established glycosylation procedures. Two additional compounds **144** and **145** being 6’-NH_2_ analogues of **129** and **137** respectively, were also prepared in order to solve solubility problems. Emerging from this series as a potential clinical candidate was compound **145** which exhibited *in vitro* topoisomerase I mediated cleavage activity and topoisomerase I selective cytotoxic profile with improved solubility and pharmacokinetic behaviour. In continuation of this work and considering that the introduction of a 3,9-difluoro substitution pattern on the indolocarbazole core confers topo I selectivity, the same group proposed a new series of analogues similarly fluorosubstituted in the carbazole framework but also fluoroglycosylated [63]. The introduction of fluorine into the 2’, 4’ and 6’ positions of the sugar portion of the molecule was accomplished both pre- and post-glycosylation. From this series, compound **146** displayed a broad spectrum antitumor activity (superior to CPT-11!) against some preclinical xenograft models, including curative antitumor activity against Lewis lung carcinoma, and was consequently chosen as a lead clinical candidate (Table 2).

**Figure 13.**
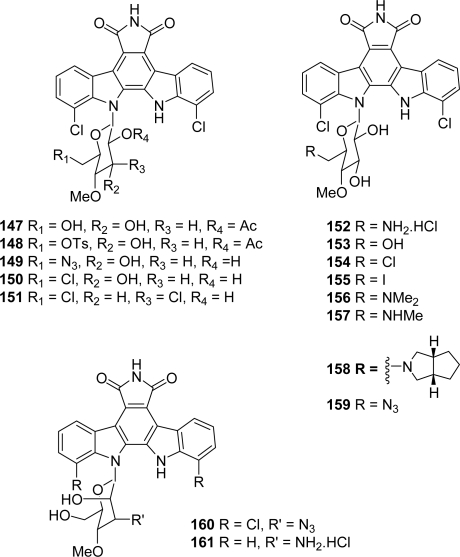
Rebeccamycin analogues bearing different substituents on the sugar moiety.

Since the sugar moiety is often the critical determinant of the key biological activity of indolo[2,3-a]carbazoles, inhibition of protein kinases for compounds containing two glycosidic bonds (e.g., staurosporine) or DNA-binding and antitumor properties for compounds with only one glycosidic linkage (e.g., rebeccamycin), this element of structure has been extensively investigated. In 2003, Anizon *et al.* reported a chlorinated (**147**–**151**, **160**) and a dechlorinated (**152**–**159**, **161**) series of rebeccamycin analogues bearing diverse substituents on the sugar moiety [64] (Figure 13).

Their interaction with DNA and their effects on human DNA topoisomerases I and II were studied. The incorporation of a 6’-amino group reinforces the capacity of the drugs to interact with DNA but almost abolishes their poisoning effect on topoisomerase I suggesting that DNA and topoisomerase I represent two independent targets.

**Scheme 12.**
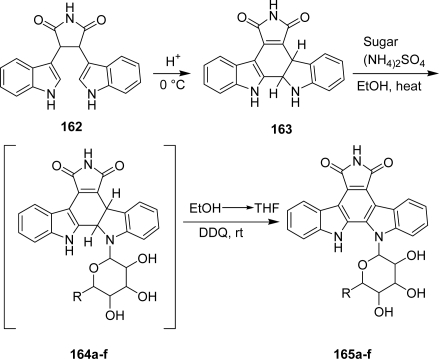


Another series of pyrrolocarbazole-fused indole analogues that lack the aryl chlorine group and in which the 4-*O*-methylglucose moiety has been replaced by a naturally occurring sugar including d-glucose (**165a**), d-galactose (**165b**), l-fucose (**165c**), l-rhamnose (**165d**), d-xylose (**165e**) and d-maltose (**165f**) was described by Faul *et al.* [65]. These compounds were prepared according to an efficient two-step process via the indole-indoline intermediate **163** which was obtained by performing at 0 °C an intramolecular Mannich process starting from precursor **162**. Compound **163** was glycosylated and the resulting products were oxidized in a one-pot procedure (Scheme 12).

**Figure 14.**
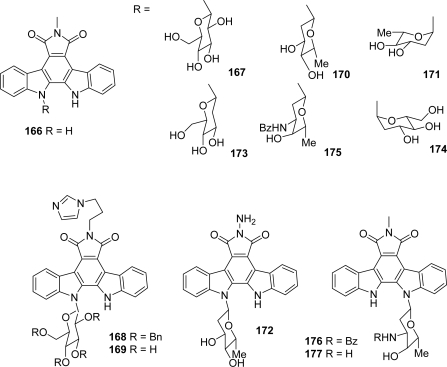
Rebeccamycin analogues with uncommon sugar.

Compounds **165a**–**f** were evaluated for their D1-CDK4 inhibitory activity. In addition, other assays such as B-CDK1, E-CDK2, PKA and CAM II were also conducted to determine their selectivity profile. All the compounds demonstrated good inhibitory activity for D1-CDK4 and they also showed improved selectivity toward several other kinases, the l-rhamnose derivative **165d** being found to be the most selective and potent analogue for D1-CDK4 (IC_50_ = 76 nM). More recently, Zhang and co-workers investigated rebeccamycin analogues containing uncommon sugars [66]. They proposed four groups of compounds **167**–**177** depending on the substituent on the imide nitrogen (Figure 14).

Their cytotoxicities against colon cancer and leukaemia cells together with their ability to target topoisomerase I were examined. Compared with the aglycon **166**, the modified compounds showed more potent cytotoxicities and topoisomerase I targeting ability. The better activities of compounds **167**, **173**, **169** and **174** imply that the 2- and 6-OH groups may have a more significant role than other OH groups in the sugar unit. In addition, the cytotoxicities of these compounds clearly correlated with the inhibition of topo I suggesting that the sugar moiety, especially the 2- and 6-positions, is a key element for the activity.

**Figure 15.**
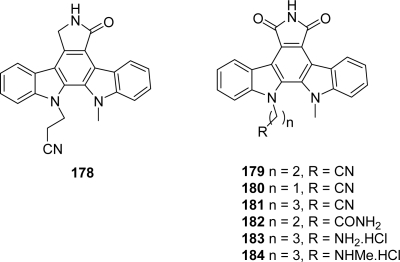
Rebeccamycin analogues substituted on the indole nitrogens.

During their work to identify novel potential inhibitors of Chk1 related to the indolocarbazoles **178** and **179** [67], Gribble *et al.* have synthesized and tested two new nitrile homologues **180** and **181** and an amide analogue **182** [68] (Figure 15). This series of compounds can be considered both as granulatimide analogues in which the imidazole ring has been replaced by a second indole ring or as rebeccamycin analogues in which the sugar part was replaced by a functional chain.

**Scheme 13.**
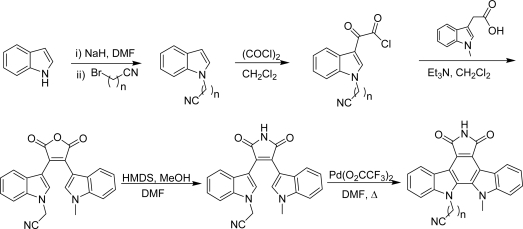


All the compounds were obtained by adapting the methodology previously described by Faul *et al.* [69,70] in which the construction of the bisindolyl maleimide was realized by condensation of indole-3-acetamide with methyl indole-3-glyoxylate (Scheme 13).

In the case of compound **182**, the amide-chain was attached on the indole-3-acetic acid fragment in order to circumvent solubility problems. In an assay using flow cytometry analysis, compounds **180**, **181** and **182** were found less potent (3 μM, 10 μM and 1–3 μM respectively) than compound **179** (100 nM) at abrogating DNA damage-induced cell cycle arrest. These values were compared to the efficiency of **178** of 30 nM in the same assay. From these results, it was found that a three-carbon nitrile chain provided maximum activity and that the cyano group was a more desirable functionality than the amide.

In the course of their structure-activity relationship studies, by using the same methodology, the same group then prepared the two hydrochloride compounds **183** and **184**, as other amine-analogues [71]. Compound **184** was proved to abrogate S-phase arrest at 100 nM indicating that it inhibits Chk1 whereas compound **183** was found inactive in the same flow cytometry assay.

**Figure 16.**
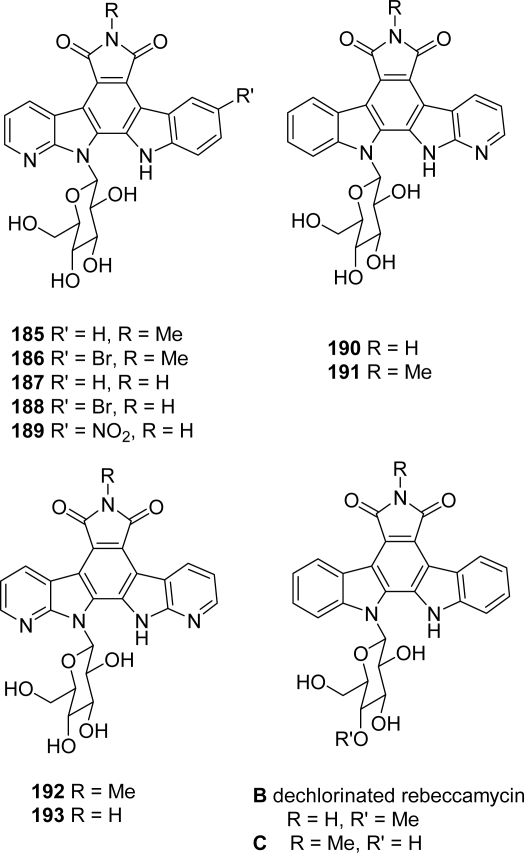
7-Azarebeccamycin analogues bearing one or two azaindole moieties and dechlorinated rebeccamycin analogues B and C.

One of the modifications in rebeccamycin structure previously described by Routier and cols. [72,73], but since extensively studied by Prudhomme and her group, is the bioisosteric replacement of an indole moiety by a 7-azaindole unit [74]. They have first been interested in a series of compounds **185**–**191** containing one azaindole unit with or without a methyl group on the imide nitrogen and with the sugar moiety linked either to the indole nitrogen or to the azaindole nitrogen (Figure 16).

In a second series of analogues **192**–**193**, the two indole moieties of rebeccamycin were replaced by two aza-indole units (Figure 16). The compounds were synthesized according to strategies already described. Depending on the expected product, the sugar part was introduced before or after the cyclization which formed the central benzenic ring (*i.e.*, unit C). In the case of post-glycosylation approach, the substitution of the 9-indole hydrogen was realized in the last step. The DNA-binding and topoisomerase I inhibition properties of the new compounds were investigated together with the antiproliferative activities toward nine tumor cell lines and their effects on the cell cycle of L1210 leukemia cells. In contrast to their non-azaanalogues (rebeccamycin, dechlorinated rebeccamycin **B** and compound **C**), which were cytotoxic for all the cell lines tested, the azaanalogues showed a selective action toward certain cell lines in the nanomolar range. All compounds induced similar cell cycle effects, with a marked G2+M block observed with L1210 leukemia cells. In addition, the sugar unit linked to the indole moiety promoted higher affinity for DNA whereas compounds with the sugar linked to the azaindole moiety have lost their DNA-binding affinity. In these series, a good correlation between DNA binding and topoisomerase I inhibition was observed. These data suggest the possibility of targets other than DNA and topoisomerase I for the azaanalogues.

**Scheme 14.**
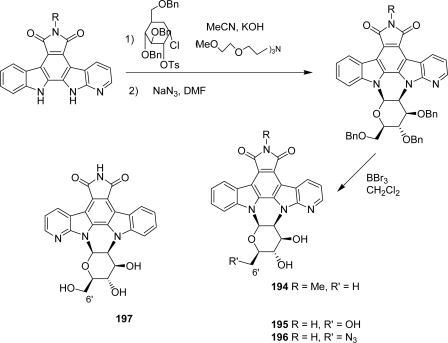


The next step in the investigation of 7-azarebeccamycin related compounds was the study of bridged azaanalogues, namely compounds with both indole and azaindole moieties linked to the carbohydrate residue (Scheme 14).

**Figure 17.**
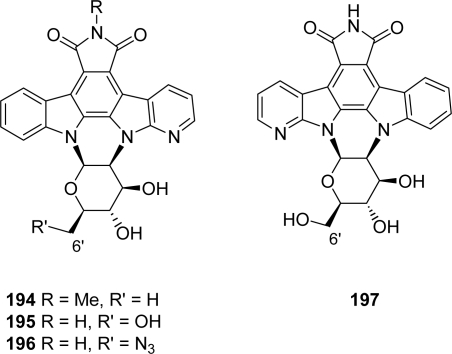
Bridged aza-rebeccamycin analogues.

A previous report described the preparation of compound **194** which could be achieved by coupling a chloro sugar moiety previously tosylated in 2’-position to the corresponding azaindolocarbazole aglycone and subsequent reaction with sodium azide in DMF [75] (Figure 17). A series of compounds **195**–**197** bearing a free imide nitrogen and in which the oxygen of the pyranose heterocycle is oriented toward either the indole or the azaindole unit was then proposed [76]. The same methodology was applied to synthesize the derivative **196** using a 2’-tosylated instead of a 2’-chloro glycosyl donor. No data on cytotoxicity and inhibitory activities of these compounds towards various kinases are available so far.

**Figure 18.**
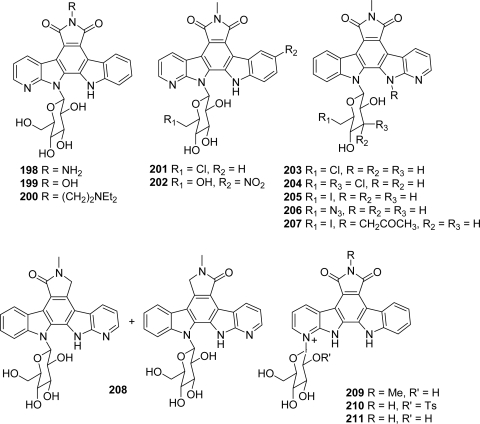
7-Aza-rebeccamycin analogues with various substituents on the sugar moiety, on the imide nitrogen, on the carbazole framework or on the nitrogen of the pyridine ring.

In the next proposed series, the modifications have been carried out together on the upper heterocycle (introduction of substituents on the imide nitrogen or replacement of the imide by a lactam function), on the carbazole framework and the sugar part, this last unit being linked either to the azaindole moiety (**198**–**202**) or to the indole (**203**–**208**) [77] (Figure 18).

The cytotoxicities of these compounds toward four tumor cell lines including L1210, A549, DU145 and HT29 showed that compared with the parent compounds, the modifications on the new analogues are not detrimental to the *in vitro* activities. As shown in the case of previous series, when the sugar part was linked to the indole moiety, the affinity for DNA and topoisomerase I inhibition were highly enhanced compared with non-aza parent compounds whereas, when the sugar was attached to the azaindole, the affinity for DNA and topoisomerase I inhibition were highly decreased. Nevertheless, strong cytotoxicities were observed toward certain cell lines, probably due to other targets.

In the last series of azaanalogues examined by Prudhomme’s group **209**–**211**, the sugar moiety was linked to the nitrogen of the pyridine ring [78] (Figure 18). These products were obtained as byproducts in the course of the synthesis of 7-azaanalogues, precisely during the final Mitsunobu-type glycosylation step. The binding to DNA appeared enhanced in the case of these new compounds. However, as observed with other rebeccamycin analogues, the DNA-binding affinity was not correlated with topoisomerase I inhibitory properties. It seemed also, from a Chk1 inhibitory point of view which was also examined in this series, that the shifting of the sugar from the nitrogen of the indole to the nitrogen of the pyridine increased the effect.

## 5. Granulatimide Analogues in Which the D and E Rings Are Modified or Absent

**Figure 19.**
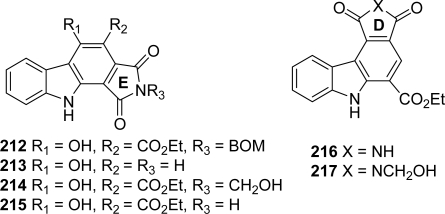
Granulatimide analogues in which the D and E rings are modified or absent.

In the course of structure-activity relationship studies on granulatimide as Chk1 inhibitors, substituted pyrrolocarbazoles **212**–**215** in which the upper imide-type unit D is missing and pyrrolocarbazoles **216** and **217**, in which the imidazole unit E is missing were investigated [79] (Figure 19).

The compounds of the first series were synthesized via compound **212** itself obtained by reaction of ethyl-3-oxo-(1H-indol-3-yl)propionate and N-BOM-dibromomaleimide whereas the derivatives of the second series were prepared on the basis of a Diels-Alder reaction between indolylmaleimide and ethyl acrylate. Concerning biological activities, it appeared that compounds in which the upper unit D was missing, were poor Chk1 inhibitors suggesting that this ring is required for Chk1 inhibition. In contrast, the lower heterocyclic unit E is not absolutely required for Chk1 inhibition. Moreover, it seems that substitution of the imide nitrogen with a hydroxymethyl group was not detrimental to Chk1 inhibitory activity.

## 6. Conclusions

Most of the granulatimide derivatives clearly represent a promising class of antitumor agents due to their potent inhibitory activity against different kinases. Recent reports on indolocarbazole analogues have shown that minimal structural modifications are able to modify both the biological targets and induce selectivity towards tumor cell lines. For instance, substitutions in 3,9-positions of the rebeccamycin framework may induce strong inhibitory properties toward various kinases and selectivity toward the tumor cell lines. The same observation is also valuable in the case of pyrrolocarbazole analogues which means that it could be of interest in these carbazole series to evaluate systematically the different compounds toward a large panel of kinases, such a profile of kinase selectivity being obtainable thanks to modern high-throughput technologies [80,81].

## Figures and Tables

**Table 1.** PARP *in vitro* activity of pyrrolocarbazole.
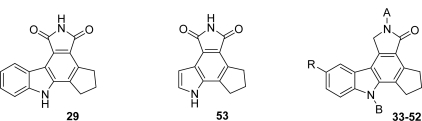

**Compound**	**A**	**B**	**R**	**PARP-1 IC_50_ (nM)**
**29**	-	-		36
**33**	H	H	H	56
**34**	H	H	Cl	120
**35**	H	H	Br	30
**36**	H	H	CN	18
**37**	H	H	CH_2_NH_2_	27
**38**	H	H	Me	200
**39**	H	H	CO_2_H	80
**40**	H	H	CO_2_Me	59
**41**	H	H	CONH-(CH_2_)_2_-NMe_2_	165
**42**	H	H	CONH-(CH_2_)_2_-morpholin-4-yl	162
**43**	H	H	CO-morpholin-4-yl	83
**44**	H	H	CON(Me)-CH_2_-pyrid-4-yl	65
**45**	H	H	CON(Me)-CH_2_-pyrid-2-yl	237
**46**	H	H	CON(Me)-(CH_2_)_2_-imidazol-4-yl	161
**47**	H	H	CONH-(CH_2_)_2_-triazol-1-yl	105
**48**	H	H	CH_2_NHCOCH-(NHBoc)[(CH_2_)_4_NHBoc]	670
**49**	H	H	CH_2_NHCOCH-(NH_2_)[(CH_2_)_4_NH_2_]	80
**50**	H	Me	H	800
**51**	Me	Me	H	10,000
**52**	CHO	H	H	3,000
**53**	-	-		40

**Table 2.** Structure-*in vitro* activity relationships for some indolocarbazole analogues against human topoisomerase I and murine P388 leukemia cells.
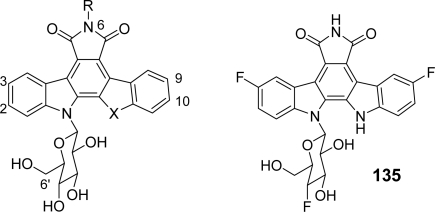

**Compound**		**X**	**R**	**Topo^a^**	**P388^b^**	**R/S^c^**
	rebeccamycin	NH	H	>500	0.54	1.26
**128**	2,10-diF	NH	H	2.0	0.26	8.7
**129**	3,9-diF	NH	H	0.22	0.018	182.7
**130**	2,3,9,10-tetraF	NH	H	0.69	0.007	67.1
**131**	2,3,9,10-tetraF	NMe_2_	H	>600	>8.72	>1.1
**132**	3-F	NH	H	6.6	1.036	>905
**133**	2-F	NH	H	3.1	0.392	4.6
**134**	10-F	NH	H	>200	0.098	11.4
**135**^d^	9-F	NH	H	1.7	0.101	31.7
**136**	3-F	S	H	2.2	0.155	>51.3
**137**	3,9-diF	S	H	0.09	0.010	232.5
**138**	3-F	O	H	1.5	0.529	13.6
**139**	3,9-diF	O	H	0.27	0.114	63.7
**140**	3,9-diF	NH	NH_2_	0.22	0.020	196.9
**141**	3,9-diF	NH	OH	0.08	0.035	19.6
**142**	3,9-diF	NH	Me	1.0	0.862	>10.8
**143**	3,9-diF	S	Me	0.48	0.236	14.1
**144**^e^	3,9-diF	NH	H	0.28	0.326	28
**145**^e^	3,9-diF	S	H	0.46	0.068	>115
**146**	3,9-diF	NH	H	0.11	0.002	380

a Ratio of the median effective concentration (EC_50_, μM) of compounds for inducing single-strand breaks in the DNA substrate divided by that obtained for CPT in the same experiment. CPT mean topo I EC_50_ = 160 nM.

b Mean cytotoxic concentration (IC_50_, μM) following 3 days of continuous exposure of compound to P388 murine leukaemia cells. CPT mean P388 IC_50_ = 36 nM.

c Ratio resulting from the cytotoxicity IC50 value obtained for CPT-resistant P388/CPT45 cells divided by that obtained for parental P388 cells.

d Inseparable mixture with **132**.

e Both **144** and **145** are 6’-NH_2_ analogues.
